# The After-Care of the Wounded

**Published:** 1914-09-12

**Authors:** 


### The Hospital

**The Workers' Newspaper of Administrative Medicine and Institutional Life, Administration, National Insurance and Health.**

No. 1473, Vol. LVI. SATURDAY, SEPTEMBER 12, 1914. PRICE ONE PENNY.

### THE AFTER-CARE OF THE WOUNDED.

The arrangements for the reception of the sick and wounded soldiers have now been brought to a satisfactory condition, and the organisation through the War Office is practically complete. The hospitals to which the wounded will be sent are the military hospitals, the Territorial hospitals, and those voluntary hospitals which have offered accommodation that has been accepted, so that the beds available are located all over the country. Those on the East Coast and in the Southern and Midland sections are likely to be first utilised. In the result some 50,000 beds can thus be provided, if and when so large a number is required. Everybody must hope that they will not be, but in a great war like that in which we are at present engaged it is impossible to forecast the future. The severer cases have so far been sent mainly to military hospitals. Those admitted to voluntary hospitals have consisted mainly of relatively simple cases, and it is hoped that the majority will speedily recover, and that many of them may soon be in a position to rejoin the Colours.

It is the men's wish, as well as that of the authorities, that recovery may be expedited in every case by all possible means, and it may therefore be well to say something to-day about after-care provision. We understand that the men in military and Territorial hospitals will not, it is contemplated, be sent to semi-convalescent institutions at all. It is felt that they would prefer, wherever possible, directly they attain convalescence to return to their friends rather than to go on to some other institution. This wish on the men's part is subject, of course, to the home conditions, and if they are not suitable on economical and humane grounds it is essential that arrangements shall be everywhere made to secure that every sailor or soldier on attaining convalescence who needs after-care shall find himself in a position to secure it immediately he is discharged from the hospital. In considering the nature of the provision best adapted to facilitate the ends in view, remembering that the present is our war, that is, the business of everybody, civilians and warriors alike, it is of the first importance to bear in mind that every civilian who from circumstances is unable to join the Forces is necessarily in the position of a trustee to do something personally and definitely as a grateful friend of every sailor and soldier who is fighting for freedom and peace. Mr. Bonar Law, speaking at the Guildhall on the 4th instant, properly insisted that sacrifice and patriotism are not only required in the present crisis from those who are able and willing to fight our battles, but from every man and woman resident in the British Islands and throughout the Empire. "At least equal sacrifice is demanded from those who remain behind as from those who are at the front or on the sea. It is therefore a matter of duty and privilege that as a nation each one of us should realise our obligation and make a vow, and keep it, that no dependant of any man who is fighting our battles shall go hungry while we have bread to eat. It is essential that each of us should realise, as we have not always realised in the past, that our sailors and soldiers are children of the State, and that they have the first claim upon the resources of our nation."

Mr. Bonar Law voices the sentiments of the overwhelming majority of the people of these islands. Unfortunately there is reason to fear that the spirit of Bumbledom has become active owing to the institution by the Prince of Wales of the National Relief Fund. Evidence accumulates daily that the dependants of men who are fighting our battles have gone hungry already; that they have been passed on to the Charity Organisation Society, and thence to the Poor-Law Guardians, from whom relief has been obtained. The mere statement of a fact like this is well calculated to make the blood of every honest man and woman throughout the land boil with indignation, and steps must be taken without a moment's delay to render such things impossible in the future. There is, so far as our inquiries go, little evidence to show that any exceptional real distress exists at the present time. Residents in London have been disgusted to notice that the establishment of the National Relief Fund has brought thousands of loafers to the Metropolis who may be seen in groups lounging and lying about the parks and open spaces, though the large majority of them are able-bodied men, many of them in the prime of life. It is up against the Government and the police to start these loafers back again to the places from which they came, failing their enlistment, for their presence is an insult to our common humanity in the present crisis. Indeed it must be made impossible that any one of these degenerates shall receive relief as distressed persons from the National Relief Fund.

These considerations have some bearing on the after-care of the wounded, because they emphasise how important it is that the residents in each district from which recruits have been drawn shall impose upon themselves the privilege of seeing that there is sufficient convalescent accommodation in their midst to enable every wounded man, the circumstances of whose family demand it, to obtain after-treatment and care, with the necessary feeding up and attendance which can best contribute to his speedy restoration to perfect health. We quite sympathise with what we understand to be the War Office view—namely, that the wounded convalescent would prefer to spend his furlough until complete recovery among his own people, or at any rate in the district from which he comes. In this way no doubt our suggestion would tend to bestow privileges upon his well-to-do neighbours as well as help practically and in a neighbourly spirit each of the soldiers concerned. There remain the more severely wounded and longer cases where convalescent aid involving nursing, dressings, and so forth is required. In these cases, of the numbers of which it is at present not possible to make any reliable estimate, the accommodation should be situated in some healthy, invigorating country district, where the air is good and the surroundings are attractive. It may be well in this connection to repeat the caution originally circulated by the British Red Cross Society to those who came forward with offers of houses and many other things, that it will be a waste of money to attempt to prepare any accommodation of this kind until a definite request for its provision is received from Headquarters by the intending donors.

